# Odour generalisation and detection dog training

**DOI:** 10.1007/s10071-024-01907-0

**Published:** 2024-11-01

**Authors:** Lyn Caldicott, Thomas W. Pike, Helen E. Zulch, Daniel S. Mills, Fiona J. Williams, Kevin R. Elliker, Bethany Hutchings, Anna Wilkinson

**Affiliations:** 1 School of Life and Environmental Sciences, University of Lincoln, Lincoln, UK; 2 Defence Science and Technology Laboratory, Porton Down, Salisbury, UK

**Keywords:** Generalisation, Olfaction, Odour learning, Detection dogs

## Abstract

Detection dogs are required to search for and alert to specific odours of interest, such as drugs, cadavers, disease markers and explosives. However, the odour released from different samples of the same target substance will vary for a number of reasons, including the production method, evaporation, degradation, or by being mixed with extraneous odours. Generalisation, the tendency to respond in the same manner to stimuli which are different – but similar to – a conditioned stimulus, is therefore a crucial requirement for working detection dogs. Odour is a complex modality which poses unique challenges in terms of reliably predicting generalisation, when compared with auditory or visual stimuli. The primary aim of this review is to explore recent advances in our understanding of generalisation and the factors that influence it, and to consider these in light of detection dog training methods currently used in the field. We identify potential risks associated with certain training practices, and highlight areas where research is lacking and which warrant further investigation.

## Introduction

Detection dogs have been used to assist humans in key areas such as security (detecting explosives or illegal drugs), and health (finding injured or sick people or animals, or identifying diseased human tissue) for many years, and are considered an important asset in terms of national security (Marshall and Oxley 2011; Moser et al. 2019). As a result, there is increasing interest in research investigating dogs’ ability to effectively detect and alert to different targets and the factors that may influence this (Troisi et al. 2019), particularly how different training techniques affect efficacy (Marshall and Oxley 2011; Hayes et al. 2018; Lazarowski et al. 2020).

While training a dog to search for and indicate the presence of the odour of a single specific substance but not others (discrimination) is a superficially simple process (Rutter et al. 2021), the reality is quite different due to the challenges associated with odour stimuli (Lazarowski and Dorman 2014; Moser et al. 2019; Aviles-Rosa et al. 2021; DeChant and Hall 2021). For example, the odour emitted by a target substance may vary due to factors such as the environment in which it is found, the process of ageing, the place of manufacture, or the physical quantity in which it is found (Aviles-Rosa et al. 2021; Jezierski et al. 2016; Moser et al. 2019; DeGreeff and Peranich 2021). In addition, the targets that an operational dog may be required to detect may be multiple and wide ranging; for example, an explosives detection dog may to be required to alert to devices containing any of a large number of different military, commercial and home-made explosives along with a wide range of background odours (Lazarowski and Dorman 2014; Hall and Wynne 2018; Wright et al. 2017; DeGreeff and Peranich 2021). This can result in a substantial amount of variation in the odour emitted by substances, making it impossible to specifically train a dog for all potential exemplars of the target that they may encounter. To account for this, the dog must learn not only to alert to their trained odours but also to generalise their response to similar odours.

Stimulus generalisation refers to the tendency to respond in the same manner to stimuli that vary (within given limits) from the originally conditioned stimuli (Ghirlanda and Enquist 2003; Pearce 2008). The degree of difference between the trained and a novel stimulus is the key quality that will affect the likelihood of generalisation; the greater the similarity of the stimuli, the more likely generalisation will occur (Spence 1937; Pearce 2008), although there are many additional factors that influence the process. In reality, a careful balance is required between generalisation and discrimination as it is important that there is adequate generalisation to ensure an explosive device does not go undetected, or a drugs cache does not find its way to the streets (Marshall and Oxley 2011; Lazarowski and Dorman 2014), while sufficient discrimination is maintained to ensure false alerts do not result in the unwarranted closure of events, or unnecessary detention of individuals. Currently, we lack full understanding of how odour perception, identification and generalisation is affected by training methodology (Keep et al. 2021; Caldicott et al. 2024). However, we do know that the ability to manipulate a dog’s processing of a target odour, via training paradigms and interventions, is key to influencing the breadth of generalisation to allow the dog to meet the requirements of the specific task (Mandairon et al. 2006; Marshall and Oxley 2011; Hall et al. 2015; Wright et al. 2017; Hall and Wynne 2018).

While there is much research investigating the phenomenon of stimulus generalisation within the visual and auditory domains, there is remarkably little direct evidence to draw on in terms of olfactory stimuli. Therefore, this review will first consider our current understanding of the principles of generalisation from these other domains, while highlighting the unique and varied challenges odour creates for the transference of such research. In addition, we review current understanding of the impact that different learning paradigms have on the discrimination-generalisation balance and make recommendations for the most effective methods to improve generalisation. Finally, we investigate other factors relating to exposure and experience of stimuli, which may impact odour generalisation. Throughout we highlight key next steps for research in the area.

## Generalisation

**Fig. 1:**
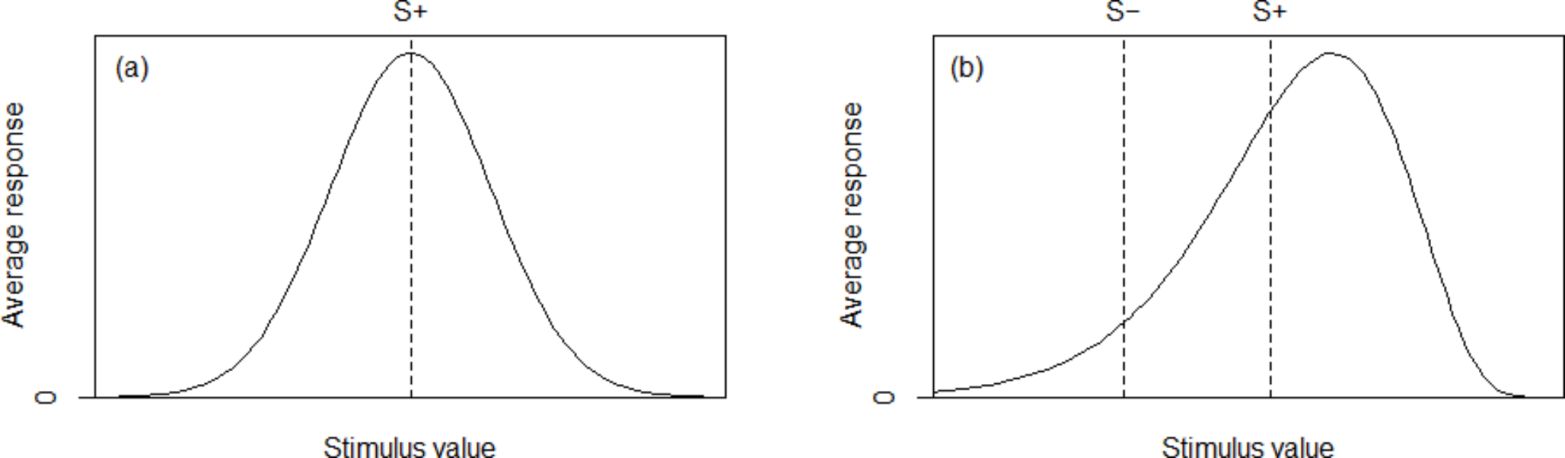
Generalisation curves. The curve in (**a**) demonstrates a symmetrical generalisation curve, resulting from training with one S + only. The curve in (**b**) shows the effect of discrimination training, training with an S + and an S– and the resulting ‘peak shift’ which has shifted to the right of the point of the S+, away from the S–

Generalisation is traditionally visualised in terms of a Gaussian curve (see Fig. 1) depicting the probability of responses to the target stimulus (S+) and variations either side of the S+, indicating the breadth and strength of generalisation (Pearce 2008). A narrow curve indicates increased discrimination (or lack of generalisation) and a broad curve increased generalisation (or lack of discrimination; Pearce 2008). The steepness of the side of the curve depicts the distinction made between what is, and what is not, considered an S+; the steeper the slope the more clearly demarcated the S+.

The generalisation curve for a learned S + is typically symmetrical with the peak of responses occurring at the location of the S+ (Fig. 1a). However, when discrimination learning is applied to this through the introduction of a non-rewarded stimulus (S–), the peak of responding no longer occurs at the point of the trained S+ (Pearce 2008). When the stimuli being considered vary along a continuous linear scale (such as colour, sound or light), the peak of responses shifts from the initial maximal point of the S + to a stimulus that is further removed from the S– on the linear scale (Fig. 1b). This phenomenon is known as “peak shift” (Spence 1937; Dougherty and Lewis 1991; Perez et al. 2016). Several factors relating to the stimuli are known to affect peak shift. For example, the physical difference between the S + and the S– is known to affect the magnitude of peak shift, such that the greater the perceived difference between the S + and S– the less peak shift will occur in relation to that metric (Ghirlanda and Enquist 2003; Wisniewski et al. 2011; Pearce 2008). Peak shift is also impacted by the number of S + and S– provided during training; for example, the peak of responding can be manipulated by providing two S–, one either side of the S+ (Dougherty and Lewis 1991; Lazareva et al. 2005; Pearce 2008). The closer the S– are positioned along the linear scale to the S + the narrower the curve will become (i.e. increased discrimination). Peak shift is an important consideration in all discrimination/generalisation paradigms, as if the peak of responding is significantly removed from the original point of the S+, the level of generalisation to stimuli that lie between the S + and S– on the linear scale may be reduced.

Peak shift is a pivotal concept affecting our understanding of the mechanisms underpinning generalisation as a natural phenomenon; however, it relies on the manipulation of a clear and specific quality of the stimulus (e.g., brightness, sound waves, or colour spectrum) (Weiss et al. 2012). This is relatively straightforward to conceptualise and plot in linear form as an x-axis on a generalisation graph (see Fig. 1b) for these types of stimuli. With stimuli such as these, clear and reliable predictions can be made regarding how animals are likely to generalise to stimuli related to the S + along the specific measure of interest (Wisniewski et al. 2011). However, when considering the diversity of odours to which generalisation may occur, there is no obvious metric that can be described in this linear form (Weiss et al. 2012). Although chemical similarity (and in particular, carbon chain length and functional group) have been used to provide a linear spectrum (Linster and Hasselmo 1998; Yoder et al. 2014), research involving this metric with detection dogs has revealed conflicting results (Hall et al. 2016b; DeGreeff et al. 2020; discussed below). Thus, the basis upon which dogs perceive odours to correspond with each other in terms of similarities or differences is still unclear (Trimmer and Mainland 2017; Thomas-Danguin et al. 2014). Therefore, in terms of odour, there is currently no consensus metric that can be used to reliably describe the perceptual similarity of related molecules (Weiss et al. 2012); indeed, such a relationship might simply not exist (Quian et al. 2023). This is problematic when we consider this to be the basis on which generalisation occurs, and limits the development of appropriate training aids for detection animals. This issue is confounded further when the intricacies of what constitutes an odour are considered; this is discussed in the next section.

## Fundamental principles of odour perception

The perception of an odour typically arises from the detection of a complex blend of various gaseous molecules, called odorants. When odorants are detected, they are initially processed by olfactory receptors. Each odorant may simultaneously activate multiple olfactory receptors (and different odorants may share some of the same olfactory receptors), thus creating a unique combination of neuronal signals which are processed by the mitral cells in the olfactory bulb (Buck 2005). The mitral cells integrate the signals received from the receptors and convey this information to the olfactory cortex enabling recognition and identification of the particular odorant being experienced (Jinks and Laing 1999; Buck 2005; Rodríguez et al. 2011). When odours comprise a mixture of multiple odorants, with each odorant having its own pattern of receptor stimulation, the resulting signal from the simultaneous activation of multiple receptor patterns, which may overlap, could result in the mitral ‘code’ retaining some, all, or none of the specific perceptual qualities of the individual constituent odorants (Laska and Hudson 1993; Jinks and Laing 1999; Laing et al. 1989; Thomas-Danguin et al. 2014; Trimmer and Mainland 2017; Rodríguez et al. 2011). For example, if olfactory receptors A, B, and C respond to one odorant, and receptors B, C, and D respond to another, a combination of both odorants will activate receptors A, B, C, and D. The resulting signal from the mitral cells may be able to reflect the constituent odorant, but it might, at least theoretically, mimic that of an entirely different odorant (one that also stimulates A, B, C and D) and thus be perceived as something quite different to either of the two constituent odorants. It is currently suspected that odours which overlap significantly in olfactory receptor activation are likely to be perceived as similar (Linster et al. 2001; Rodríguez et al. 2011); and while generalisation theories suggest that similar stimuli are more likely to result in the same behavioural response, when we consider odour, this may not always be the case. When similar odours are mixed, specific perception challenges are known to occur. These include masking, a phenomenon that prevents one stimulus being perceived over another (Kim 2010; Laing et al. 1989) therefore preventing identification (discrimination), and overshadowing, in which less is learned about a less salient odour than another more salient one when presented together during the associative learning process (Schubert et al. 2015). Synergistic effects, where interactions lower perceptual thresholds can also occur (Yoder et al. 2012).

**Fig. 2:**
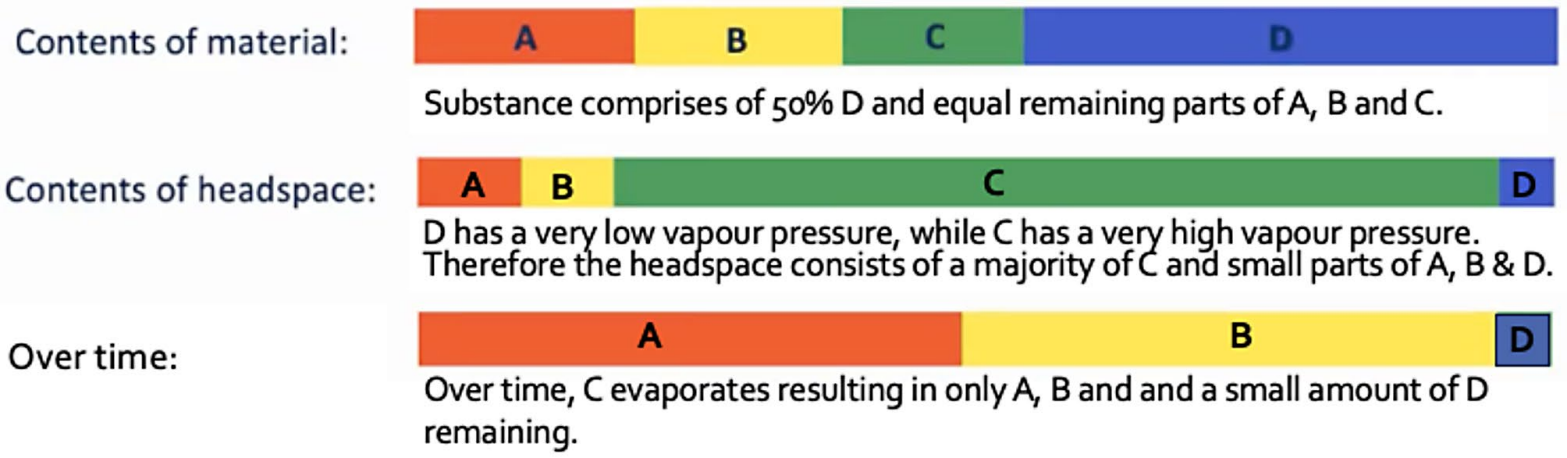
Example of how odour perception may change due to vapour pressure and evaporation

A masking effect has been observed with olfactory stimuli in several species, and it is believed that it can be caused by one of two factors. Firstly, the perceived strength (intensity) of one stimulus may prevent the perception of another. In the case of an odour, this may be because of differing vapour pressures between constituent odorants within an odour, or between a number of competing odours, or due to differences in quantities of an odour resulting in one substance emitting a much larger amount of odour than another (Moser et al. 2019; Thomas-Danguin et al. 2014; Laing et al. 1989; Laska and Hudson 1993). Vapour pressure is a measure of the rate at which odorants are released by a substance, and may change on an hourly/daily/weekly basis, in a manner, and at a rate, that is difficult to predict (National Research Council 2004; Jezierski et al. 2008; Sinding et al. 2013; DeGreeff et al. 2020; DeGreeff and Peranich 2021). For example, the temperature of a substance affects the rate at which odorants are released from it, and high vapour pressure odorants evaporate faster than others; therefore, odorants within such odours will degrade at differing rates and change the balance of odorants being emitted over time (Fig. 2).

As shown in Fig. 2, any alteration in the ratio of odorants within the odour profile may alter the pattern of activation of the olfactory receptors and therefore potentially change the dog’s ability to perceive a trained odorant or odour over and above others (Laska and Hudson 1993; Sinding et al. 2013; Thomas-Danguin et al. 2014; DeGreeff et al. 2020). Likewise, substances manufactured in different locations (or batches) may vary slightly in the combination of the constituent parts and thus again affect odour perception (Jezierski et al. 2008; Moser et al. 2019; DeGreeff and Peranich 2021). This means that a dog’s ability to discriminate and tendency to generalise a target odour could change due to a wide range of environmental factors, potentially over very short timescales.

A second potential cause of masking is where the olfactory receptor patterns become ‘distorted’. This effect can be caused by the overlapping activation of multiple similar odorants, changing the overall perception of the odour (as described above) (Rokni et al. 2014; Yoder et al. 2014; Wiltrout et al. 2003; Jinks and Laing 1999). This phenomenon is reported to increase in line with increasing numbers of odours in a mixture, presumably due to the number of olfactory receptors involved (Thomas-Denguin et al. 2014; Linster and Hasselmo 1998; Laska and Hudson 1993; Derby et al. 1996; Yoder et al. 2014; Jinks and Laing 1999). This is particularly relevant for detection dogs, as targets commonly comprise multiple extraneous substances mixed with, or found alongside, the substance of interest. With each substance emitting its own odour, and each odour containing multiple odorants (some of which may be shared between the various odours present), the challenge the dog faces is isolating the precise olfactory pattern associated with a substance of interest from the distorted olfactory pattern the mixture results in. Another factor causing distortion of the olfactory pattern is the intensity of an odour. While a high intensity odour can create a masking effect by overpowering other odours (as described above), it may also distort the olfactory pattern (and therefore the perception of the odour), due to an increased number of receptors which are engaged in line with the strength of an odour (Buck 2005; Weiss et al. 2012; Kotthoff and Norenberg, 2015). Generally, as the quantity of the substance increases the intensity of the odour increases, and, as such, a significant difference in the quantity (either more or less) of a substance, compared to the training sample, could result in a failure of the dog to detect it; an effect reported by Aviles-Rosa et al. (2021). Given that detection dogs are often trained on a single exemplar of their target odours, understanding how this practice might affect generalisation performance is an important area to consider. Whilst this odour will change over time, it is currently unclear what impact increased variability will have on task performance in the field.

Despite these complexities, animals can clearly learn to discriminate between similar odours, including those which have previously created a masking effect (Derby et al. 1996; Linster and Hasselmo 1998). This is probably the result of changes in the animal’s odour detection threshold. The odour detection threshold is a measure of the minimum amount of odour that can be perceived by an animal (Kotthoff and Norenberg, 2015). A low odour detection threshold means the odour (or odorant) is particularly potent to the individual, and usually relates to biologically relevant odours, such as food (Kothoff and Norenberg 2015; Rodríguez et al. 2011). Such odours are more likely to be perceived by the animal regardless of vapour pressure and being mixed with high pressure interferent odours (Kotthoff and Norenberg, 2015; Rodríguez et al. 2011). The odours to which an animal may have a low odour detection threshold will vary between species as well as between individuals, and are affected by prior experience (Trimmer and Mainland 2017). Humans, for example, have a low odour detection threshold for hydrogen sulphide (the odour of rotten egg), a naturally occurring gas produced by decaying organic matter. This has an obvious evolutionary benefit helping to avoid ingesting something which may cause sickness (Stevenson 2010). Persistent exposure to an odour may either reduce the odour detection threshold (sensitisation) or increase it (habituation) (Mandairon et al. 2006; Wiltrout et al. 2003; Gibson and Walk 1956; Gibson et al. 1958; Channell and Hall 1981). Why one occurs over the other is currently not well understood and there are conflicting results in the literature (see the ‘odour pre-exposure’ section). However, the effect of associative learning on an animal’s odour detection threshold is well documented (e.g. Hall et al. 2016a; Perez et al. 2016). Consistently applied reinforcement (or punishment) during, or shortly after, odour exposure (classical conditioning) is known to reduce odour detection threshold, effectively reducing the risk of masking, and increasing the individual’s ability to detect the odour in general, and within complex mixtures (Yoder et al. 2014; Wiltrout et al. 2003; Mishra et al. 2010). In addition to this, Kudryavitskaya et al. (2021) have shown in mice that when the same reinforcement is applied to multiple odour stimuli of differing characteristics (chemical or perceptual), the mitral cell response adapts. The signals generated in response to each odour, which are transmitted to the olfactory bulb to enable identification of the odour, became increasingly similar for the different odours paired with reinforcement (Kudryavitskaya et al. 2021). This process appears to be highly plastic, and the effect is reversed when discrimination between the odours is reintroduced through a change in reinforcement (i.e., discrimination training). This potential olfactory adaptation is important, as if associative learning can reduce the differences perceived between different odours in the olfactory system, generalisation of the behavioural response can be expected to be more likely to occur across a whole set of odours that are similarly reinforced during training.

It is clear that, given the persistently changing perceptual qualities of odour as a stimulus, significant generalisation is required by a detection dog for them to be successful, not only to cope with the variation within a target odour, but also the variations that arise when a target is mixed with extraneous odours. However, it is a perceived similarity between the S + and the novel stimulus that affects the likelihood of generalisation to the latter (Pearce 2008). While factors such as those discussed above provide crucial information regarding how perception of an odour may change, or be impaired, they are not able to predict with any degree of certainty which novel exemplars have any perceptual similarity to a given S+. There is a growing body of research which endeavours to expand our knowledge base relating to perceptual similarity of odours; however, there are conflicting results, which might reflect inappropriate choice in the relevant linear metric(s) upon which odour generalisation is based.

One such area of interest is the consideration of functional group and carbon chain length (Yoder et al, 2014; Linster and Hasselmo 1998; DeGreeff et al. 2020). Hall et al. (2016b) tested odorants with the same functional group (a hydroxyl group, –OH), but with differing carbon chain length in detection dogs, and reported that generalisation to novel target odorant mixtures decreased as the carbon chain length of novel odorants increased. This suggests odorants with the same functional groups of similar carbon chain length may be perceived similarly. In a further study, DeGreeff et al. (2020) considered carbon chain length and functional group. They reported a *tendency* to generalise to odorants with a similar carbon chain length and the same functional group; however, which odorants the dogs generalised to, was not consistent. Furthermore, training on additional test odorants from the set was seen to *reduce* generalisation performance, indicating additional training enhanced discrimination rather than promoted generalisation. When testing different functional groups with a similar carbon chain length, DeGreeff et al. (2020) also reported generalisation occurred across some, but not all, functional groups. However, when functional groups differed, the dogs’ ability to generalise did improve with additional training on a second compound from the test set. In contrast to both aforementioned studies, there are also several reports of ‘similar’ odours (based on carbon chain length) producing a masking effect and preventing associative learning taking place to a target odour contained in a mixture (Jinks and Laing 1999; Wiltrout et al. 2003; Rokni et al. 2014). The reason for these contrasting results is currently unclear; however, it does suggest that there are additional factors (other than a specific feature relating to the molecular structure) of odours and odorants that affect perception (Kotthoff and Norenberg, 2015).

It is evident that we currently do not have the required understanding to reliably predict perceptual similarities and differences across odours (Kotthoff and Norenberg, 2015). It should be noted that the majority of laboratory research utilises individual odorants found within substances of interest. However, for working dogs, the substances of interest usually consist of a mixture of odorants, which can change and fluctuate with circumstance. As such, translation of current research findings to practice should be done with caution (Thomas-Danguin et al. 2014).

## Learning and discrimination

Training for detection work most commonly requires the dog to learn multiple target odours and generalise from each of the learned targets as required. The initial training of any target odour will comprise associative learning and discrimination training; the manner of implementation of each of these may affect generalisation. As outlined in the previous section, it is typically not practical to train a dog with all the individual stimuli necessary to encompass variation in the S + the dog may encounter in the real world. Clearly stimulus generalisation is essential for performance in the field, but, as explained above, how this occurs in practice remains uncertain. However, despite gaps in our scientific knowledge, it is evident that current training methods provide good results in relation to discrimination (Williams and Johnston 2002; Furton and Myers 2001), although few studies have assessed how they impact generalisation; those that have, found generalisation was limited (Aviles-Rosa et al. 2021; Johnen et al. 2017; Elliker et al. 2014). Examining how animals generalise using current methodologies may help us develop practical adaptations that improve the efficacy of both training and performance. This, in addition to robust research testing approaches which are likely to improve generalisation, is an essential future step, and relevant work to date is summarised below.

## Discrimination training

Within a practical setting, discrimination training (i.e., training the dog to learn which substances are relevant and result in reinforcement, and which do not) is a crucial stage of the training process for any detection dog. This process is relatively easy to facilitate and generally achieved through associative learning: offering the dog choices between a target odour (S+) and other extraneous odours (S–) and using differential reinforcement to associate the target odour with the provision of a reinforcer, such as a toy or food.

However, while associative learning and the resulting decrease in the odour detection threshold will assist in the discrimination of the odour of interest and reduce the risk of masking post learning, excessive associative learning trials may be counterproductive in terms of generalisation (Ghirlanda and Enquist 2003). During associative learning, every exposure to a stimulus results in a progressive optimisation of the generalisation-discrimination curve, to mirror the variation in the S + encountered (Cleland et al. 2009; Ghirlanda and Enquist 2003). Therefore, prolonged associative learning (through discrimination training) on one exemplar of the odour of interest may result in an increase in discrimination, and a reduction in the animal’s tendency to generalise to relevant variants of the stimulus (Cleland et al. 2009). This is a key point to consider in detection training protocols and does not appear to have received much attention in the working dog literature. Using variations of the target odour (e.g. different samples or quantities) or presenting the target odour alongside or in close proximity to a variety of interferent odours is recommended to overcome this issue, with a view to increasing the breadth of the generalisation curve (Wright et al. 2017; Hall and Wynne 2018). When presenting such odours concurrently care needs to be taken not to contaminate the S + with interferent odours both for odour sample integrity and safety. However, the level of variation provided in training, and the resulting effect on generalisation does not appear to have been tested experimentally in working dogs. This may be partially explained by the fact that samples of illegal, or potentially dangerous, substances of interest for training are limited and so expanding these resources significantly may be challenging. Despite this, understanding the effect of the breadth of variation in the target odour during training, and the subsequent effect on generalisation, particularly across different stimuli, is important to inform appropriate and effective training methodologies and for developing training appropriate training aids.

It is typically following an initial process aimed at discrimination that attention turns to generalisation and the ability of the dog to respond to variations of the target odour. However, it is likely that the very process of providing choices, or at least which choices are provided in these early stages of learning, will affect generalisation. Exactly how this applies to odour detection remains uncertain, but there is value in examining these phenomena in relation to the experimental literature involving other stimulus modalities.

As discussed above, discrimination training of visual and auditory stimuli can result in peak shift (for reviews, see Spence 1937; Dougherty and Lewis 1991; Perez et al. 2016; Moser et al. 2019). As previously described, in peak shift the S– used during learning will hinder generalisation to stimuli that are perceived to fall between the S– and S+. The degree to which the S + or S– are perceived as similar or different, affects subsequent generalisation tendencies, such that the generalisation curve may widen with a greater perceived difference between stimuli (causing less of a peak shift effect) or it may narrow (increased discrimination) when the stimuli are more similar (Spence 1937; Ghirlanda and Enquist 2003; McCoy and Yanko 1983). In detection training, whilst some distractors/interferents (S–) are considered extremely important, such as the inclusion of a blank or controls for plastic gloves and packaging, the rest are generally arbitrarily selected from common household substances that the dogs may come across in the real world and be required to ignore. While these may be based on items found in the environment, which the dog is required to ignore in practice, there is scope for further understanding how their inclusion influences the response to the S+. This arbitrary variability makes it challenging to predict what is actually learned, and may contribute to ‘unexplained’ failures in the dog’s performance later in training or testing. To our knowledge this has not yet been explored experimentally and is an important area for further research. Understanding the ramifications of using arbitrary distractor stimuli, and the implications of using carefully chosen S– stimuli during training could provide the basis for simple adaptations to current methods that may improve generalisation performance.

## Training methods

**Fig. 3:**
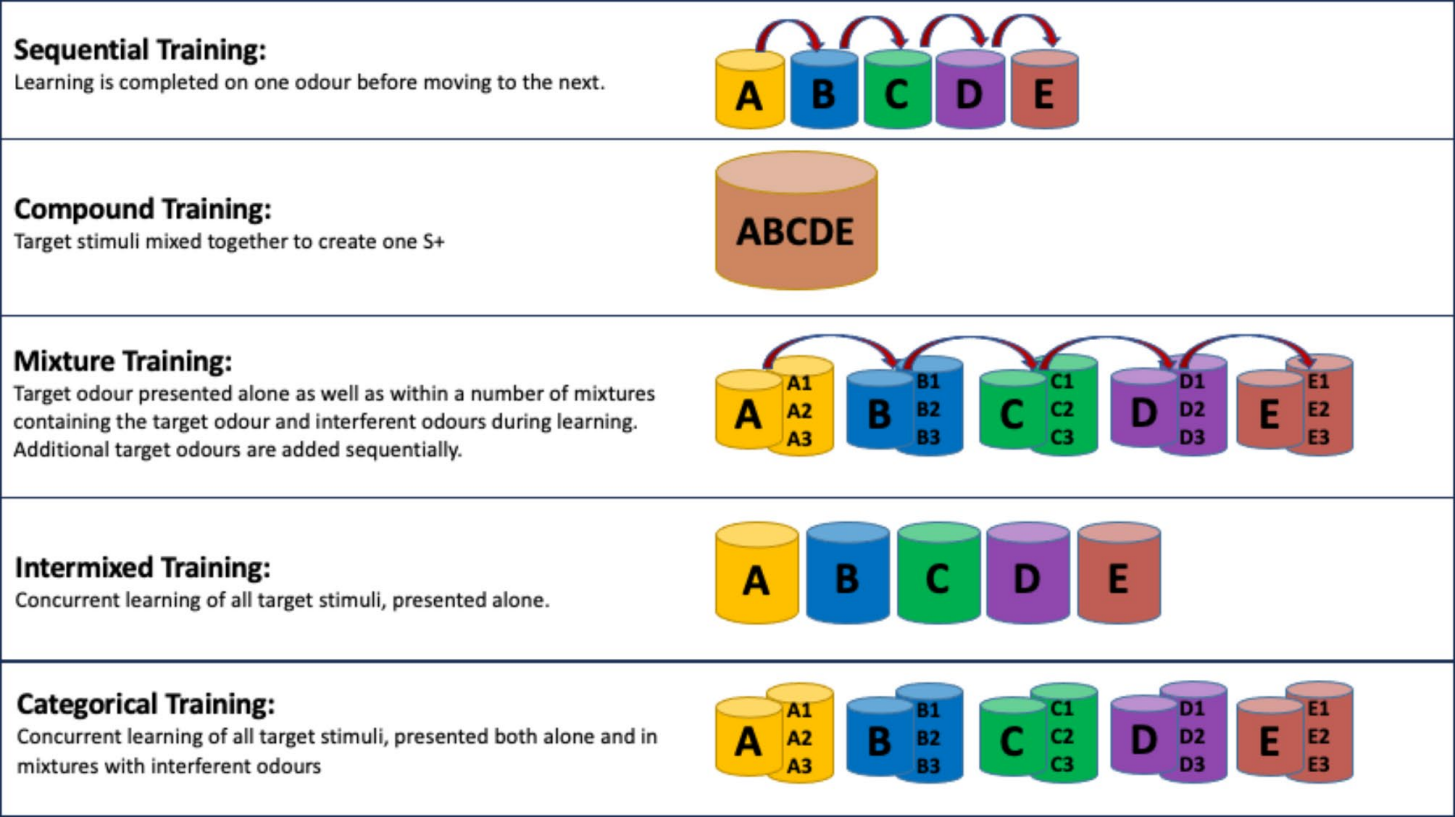
Graphical representation of common training protocols

While associative learning and discrimination training are fundamental cornerstones for all detection training, there are also various protocols which, while utilising these principles, differ in the way that the target odour is presented. The primary protocol procedures of interest, which are discussed below are: sequential training, compound training, mixture training, intermixed training, and categorisation training (Fig. 3).

### Sequential training

Currently, the most common method used for multiple odour target detection training in dogs is sequential training (for an example of common training methods see the supplementary information of DeGreeff and Peranich 2021). Here the target stimuli are presented individually, and learned by the dog one at a time, adding additional target odours only after learning on the previous odour is complete. Despite much research reporting the effectiveness of the learning of individual target odours using this method (Staubli et al. 1987; Lazarowski et al. 2021; Waggoner et al. 2022), the resulting generalisation to variations of the target odour is less effective, when compared to other methods (Keep et al. 2021; Caldicott et al. 2024). A lack of variation within the S + during training can be expected to narrow the generalisation curve, as previously discussed (Cleland et al. 2009; Moser et al. 2019). While some natural variation in the odour of the target substance will occur, due to the environmental changes and degradation effects described previously, this is unlikely to provide a broad enough variation in the target odour to effect optimum generalisation for the majority of detection tasks. To combat this effect, providing the dog with multiple variations of the target odour(s), including altering the amount of odour that is released (i.e., varying the quantity of odour), using different batches/manufacturers of the same substance, and mixing the headspace of the target substance (the odour being emitted) with odour from an interferent substance (with consideration to safety aspects of the substances in question) will provide a broader experience of the how the target odour may be represented (Wright et al. 2017) in order to facilitate generalisation.

### Compound training

A compound method (also known as a ‘stew’ method) provides multiple target odours combined as one stimulus; as such it has the potential to significantly reduce training time (Gazit et al. 2021). However, this is risky given that the substances which dogs are required to detect will vaporise and degrade at different rates, depending on environmental factors such as temperature (DeGreeff and Peranich 2021). As described above, an equal mix of substances will not necessarily provide an equal mix of detectable odorants within the headspace, and that this may change over time (Phelan and Webb 2002). Thus, in an odour stew (e.g. AB), if odour A has a higher vapour pressure it might account for the majority of the odour profile in the headspace; however, if odour B has a low odour detection threshold, then B may be perceived over A (DeGreeff and Peranich 2021). Ultimately, any mixing of target odours potentially poses a high risk for overshadowing, where the perception of a target odour is prevented during the learning phase due to the same effects of vapour pressure, odour detection threshold and the overlap of olfactory receptor activation, as previously discussed in relation to masking (Moser et al. 2019). For a stew of target odours it is not possible, at least in practice, to predict if the dog will direct their attention to the compound odour profile as a whole, or to a specific odorant (or combination of odorants) contained within it, or if one target odour will have an overshadowing effect and prevent one or more of the target odours being perceived during the learning process. Any resulting discrimination and generalisation will relate only to the dog’s perception of the odour stew, and therefore, not necessarily the individual target substances (Keep et al. 2021; Caldicott et al. 2024). Should the dog’s perception of the odour stew not be as expected, the dog may end up being trained to one odorant, a combination of odorants, or an odour profile that is not representative of the individual targets (Lazarowski and Dorman 2014; DeChant and Hall 2021).

Ultimately, the dog’s perception of an odour stew will be affected by multiple factors, none of which are easy to measure or accurately predict in the field. Although we know that dogs are capable of identifying individual odours (or odorants) from an odour compound, compound training does not facilitate the reliable processing of the component parts of the compound, and thus effective generalisation during training cannot be reliably predicted. Therefore, this method poses some fundamental questions regarding its efficacy and appropriateness as a training method and for creating the desired generalisation.

### Mixture training

Mixture training involves presenting the target odours combined with interferent (non-relevant) odours; either through mixing the headspace or through mixing the substances themselves, dependant on the safety considerations of the substances themselves. Through the process of discrimination training, this method requires the dog to extrapolate which odorants, or combination of odorants within the mixtures, reliably result in reinforcement (Hall and Wynne 2018; Wright et al. 2017; Rokni et al. 2014; Wiltrout et al. 2003; Linster and Smith 1999). As previously stated, multiple odorants often make up both a target substance odour profile and also the odour profile of the non-target substances. There is thus the potential for some of these to overlap. As with compound training, given this complex mix of odorants, such a combination may be perceived as an entirely different odour to the individual odours (or odorants) contained within it, and if not appropriately considered, the interferent odours used in target mixtures may overshadow the target odour completely, preventing effective learning from taking place (Thomas-Danguin et al. 2014; Linster and Hasselmo 1998; Laska and Hudson 1993; Derby et al. 1996; Yoder et al. 2014; Jinks and Laing 1999). To mitigate against this, mixture training not only requires target odour mixtures to be presented during learning, but also the target odour (S+) in isolation and the interferents used in the target mixtures as non-rewarded stimuli (S–). This provides the dog with meaningful comparisons from which to extrapolate an accurate representation of the target odour. As such, this process requires the dog to focus their attention on the specific properties (i.e., the odorants) that predict reinforcement, lowering the odour detection threshold for the individual odorants of importance. If the dog successfully learns each odorant within the target odour, the resulting reduced odour detection threshold to each of these odorants should enable the dog to perceive these elements amongst a variety of different and novel distractors; that is to say, generalisation is predicted to be significantly increased (Rokni et al. 2014; Hall and Wynne 2018; Kudryavitskaya et al. 2021). However, more investigation into the use of mixture training is required to fully understand the variation necessary for optimum generalisation.

Given the increased complexity of the mixture training procedure for the dog, it is perhaps unsurprising that the current literature indicates that this method may take longer for dogs to reach criteria, compared to a standard sequential procedure (Keep et al. 2021; Caldicott et al. 2024). In addition, presenting the target odours alongside interferent odours could increase the risk of contamination; rendering that sample unreliable for further discrimination training. However, careful consideration of the S– used could assist in mitigating this effect, but further research would be required to assess this. These factors have the potential to be a significant barrier to the implementation of a mixture method for detection training in practice, as training time is an essential cost/benefit consideration. However, given the improved generalisation that is likely to occur this may be a worthwhile trade-off, particularly for operational dogs that encounter a large variation in their target odours.

### Intermixed training

Intermixed training involves all target odours being learned individually but concurrently (Keep et al. 2021; Caldicott et al. 2024). This procedure has been borrowed from the methods commonly used in visual discrimination learning and is dependent upon the principles involved in the learning processes underpinning generalisation, as discussed above (Watanabe 1997; Huber et al. 2005; Kirkpatrick et al. 2007; Range et al. 2007). Intermixed training presents the dogs with multiple different target odours as individual stimuli, but requires all (or many) of the different target odours to be experienced by the dog within each learning session (Keep et al. 2021). Interferent odours are introduced from the beginning of the training (i.e. to facilitate discrimination training), thus the dog is provided with multiple odours, both target and non-target, for comparison during the learning process. Compared to mixture training, this procedure is likely to be less cognitively demanding, but it retains a wide breadth of variation in odour exposure due to the concurrent presentation of both multiple target and non-target odours. In addition, this method should eliminate the risks associated with overshadowing during the learning process that are inherent with a compound and possible with a mixed approach. Keep et al. (2021) initially investigated this approach using rats, and found that rats trained with an intermixed approach generalised significantly better than rats trained using either sequential or compound training. This process was also recently replicated in pet dogs (Caldicott et al. 2024) where, following learning, generalisation was tested in the same manner as Keep et al. (2021), through mixing the trained odours with a novel interferent odour. The dogs’ performance mirrored those found in Keep et al. (2021) such that the dogs receiving intermixed training generalised significantly better than those trained with either sequential or compound training methods.

The exact mechanisms behind the increased performance conferred by intermixed training are as yet unknown. However, being able to compare multiple S + and S– within each session facilitates the dog’s attention being drawn to the general properties of S + stimuli and likely increases attention towards similarities which is less likely and substantially more difficult in sequential training (see also categorisation training below). This is a novel technique and further investigation of an intermixed approach is required. For example, the impact that stimulus number may have on this training technique remains unclear. Whilst testing a different question, recent work by Dorman et al. (2021) found no difference in generalisation when comparing performance of dogs trained with two ammonium nitrate variants or six. However, performance overall in this study was unusually low, with multiple dogs removed from the study due to a lack of engagement in the task, and performance on trained S + dropping below criterion during the testing stage so further work is needed. Findings so far suggest it is a potentially effective and practical solution to improve generalisation. In contrast to the complexities of mixture training this method requires only a simple adaptation to practical methods that are already used in general practice.

### Categorisation training

Categorisation is an adaptive process which allows an animal to process the vast amount of information the brain perceives from the environment, filter out non-relevant stimuli and appropriately respond to anything that may be relevant (Pearce 2008). It involves the identification of a relationship between a group of stimuli in order to create a category. As such it does not require memorising a multitude of specific stimulus-reinforcement pairings, but rather creates an expectation of reinforcement in relation to a specific stimulus property that can be applied to both familiar and unfamiliar stimuli. It is thus highly adaptive as it allows the brain to efficiently process the vast amount of environmental information it perceives each day (e.g. what materials may or may not be edible).

Categorisation has been much studied in the visual domain (e.g. in dogs: Range et al. 2007, pigeons: Aust and Huber 2001; Huber et al. 2005; Kirkpatrick et al. 2007; and primates: Delorme et al. 2000). There are several theories regarding how categories may be formed (see Smith et al. 2016; Huber and Aust 2011 for full details). In brief, exemplar theory suggests each known member of the category is stored in memory and new stimuli are compared to each in turn. If similarities are observed, the new stimulus is considered to belong to the category. Prototype theory, in contrast, suggests the *average* of all known stimuli is stored in memory and a novel stimulus is compared to this; the tendency of the animal to generalise will affect whether the stimulus is considered similar enough to the average to be added to the category. Feature theory requires attention to be directed to a common feature, or features, that are present across all or most stimuli in the category; this may be one individual feature or a combination of features (e.g., odorants contained within an odour) which are predictive of the same behavioural response being successful (Pearce 2008). Given the complexity of odour and its constituent odorants, odour as a stimulus could lend itself to being categorised in this manner, this may provide a more reliable and efficient generalisation effect than the other methods described.

Wright et al. (2017) trained dogs using a categorical rule in which they were reinforced for the presence (or absence) of an accelerant. Control dogs without the categorical rule of accelerant versus no accelerant failed to learn the task, while the experimental dogs not only learned the task but also generalised to novel accelerants and novel accelerant mixtures. To create the categorical rule, this study used an intermixed training method in which the dogs were exposed to all 40 S + concurrently, and each S + was presented in a random order in every training session. Each S + was also presented on a variety of different substrates, which were then either burned or unburned. The dogs successfully extrapolated the specific aspects of the target odour (i.e. odorants) to create the categorical rule.

The findings of Wright et al. (2017) have important implications, as a categorisation training approach may provide a robust method to promote effective generalisation to different substances of the same class, account for the variations of odorants released by the target as a result of environmental factors and negate the effect of odour mixtures found in the real world. We currently do not know the specifics of the type and number of exemplars that are required to create an odour category and further investigations are required as this approach may take more time to train initially due to the potentially larger number of target stimuli required for the dog to learn. However, given that a well-defined category of a target substance is likely to provide more precise identification of the target, with greater generalisation as a result, whilst reducing the memory load on the dog, it is important that the approach is investigated further. Exploring the optimum number of S + required for a given substance class for effective category formation, may help to accurately assess the training time required and tailor the protocol to operational requirements.

## Other factors that may affect generalisation performance

So far, this review has considered the effect of specific training protocols on generalisation performance. This is perhaps the primary focus of odour detection dog training; however, it is not the only factor affecting generalisation. In this section we consider other important factors which are likely to impact the generalisation process, yet which would benefit from further research.

## Effects of pre-exposure / odour enrichment

Pre-exposure, or odour enrichment, is the process of exposing the animal to specific odours prior to training, without pairing the odour with a reinforcer. For example, the odour may be placed within a dog’s kennel when they are resting. Odour enrichment has yet to receive appropriate attention in relation to detection training, but, under the right circumstances, this may effectively enhance a dog’s perception of target odours and may therefore impact generalisation. In the case of visual stimuli, the literature suggests pre-exposure can decrease learning time and increase generalisation when the same stimuli are presented in a learning context at a later date (Gibson et al. 1958, Channell and Hall 1981; Mandairon et al. 2006; Blair et al. 2004). Reduced discrimination learning time has been observed in adult rats when the target stimuli (metal circles and triangles) were hung in their rearing cage from birth and presented in a learning context at a later date (Gibson and Walk 1956). The same effect was found by Channell and Hall (1981) when rats were exposed to images of horizontal and vertical lines in their home cage. When the rats were subsequently trained to respond to either the vertical or horizontal lines, it was found that they would also generalise their response from one to the other; rats who did not experience the pre-exposure condition did not (Channell and Hall 1981).

In terms of odour pre-exposure there is less research to call on, however some specific findings are worthy of note. Mandairon et al. (2006) used a habituation paradigm to test rats’ ability to discriminate between two chemically similar (but presumably perceptually different) odours. This study reported that rats who did not receive the odour pre-exposure condition, did not automatically discriminate between the two odours. However, rats who were exposed to an odour enrichment period comprising of the two odours for 1 h, twice a day (4 h apart), for 20 days, did discriminate between the odours. Interestingly, this discrimination ability was found regardless of whether the odour enrichment was provided with the same odours as subsequently tested, or with a different odour pair. These results imply odour exposure may alter subsequent odour perception, and of particular note here, to chemically-related odours.

Hall et al. (2014) used the odours of root beer, lemon, hazelnut and mint extracts to test the effect of odour pre-exposure on discrimination training in three groups of dogs: odour paired with a reward (associative learning); odour exposure with no associative learning (non-rewarded pre-exposure); and no exposure to the odour, as a control. The associative learning group outperformed both other groups, in terms of learning time and in the detection of their target odours. No difference was reported between the non-rewarded pre-exposed group and the no exposure group in terms of acquisition time. In addition, Hall et al. (2016a) found no effect of pre-exposure on detection thresholds. Unfortunately, generalisation was not tested in either experiment.

While the effect of odour pre-exposure in dogs has received very little investigation, the studies with rats suggest this may be an effective process. Additional studies are required, as this could be an efficient and cost-effective way to reduce the training time and potentially increase generalisation for operational dogs; it may be of particular interest for odour targets with low vapour pressure which can be more challenging to detect. This is a largely unconsidered area for detection dog research, which may have the potential to improve the efficiency and efficacy of training protocols even further.

## Experience

An incidental finding of some recent research suggests experience of the task of odour detection increases the likelihood of generalisation to variations of the target odours (DeGreeff et al. 2018; Barkat et al. 2011; Jraissati and Deroy 2021). Of particular note, DeGreeff et al. (2018) found that dogs concurrently engaged in operational or sports detection activities (with different odours to those used in the study), outperformed those who did not have this experience when tested with novel exemplars of their trained target (ammonium nitrate). In addition, Gazit et al. (2021) used both experienced operational and sports detection dogs, with unusual success when testing dogs’ ability to detect individual odours from a trained compound. While this may be an effect of exposure to a wide range of odour stimuli, prior experience in the detection task may indeed be important. Experience in the task may enhance the dog’s ability to extrapolate the necessary information relating to a new target odour presented in the same learning context. Understanding the effect on performance of differing experience levels, both task based and stimulus based, is an interesting area. This may not only inform training protocols on practices necessary to improve odour learning, but also potentially the order in which to introduce target odours to reduce training times and increase generalisation.

## Conclusions

Current evidence suggests that generalisation appears to be primarily affected by the similarity or difference between a trained odour and novel exemplars of that odour, though similarity cannot necessarily be predicted by chemical or physical properties, such as carbon chain length. In addition, the use of specific training paradigms can impact the likelihood of generalisation occurring, although the exact mechanisms that underpin this require much more investigation.

Odour is a complex stimulus, which may change rapidly and unpredictably, and may even be perceived differently by different individuals. Research into the perception of odours, how they compare, and how perception may change when odours mix, age, or are subjected to environmental changes is still in its infancy, although a number of recent studies have started to address these issues. This work, when progressed, is likely to assist us in better understanding the process of odour generalisation and how we can best promote it.

## Data Availability

No datasets were generated or analysed during the current study.
