# Correction: Odour generalisation and detection dog training

**DOI:** 10.1007/s10071-025-01992-9

**Published:** 2025-07-25

**Authors:** Lyn Caldicott, Thomas W. Pike, Helen E. Zulch, Daniel S. Mills, Fiona J. Williams, Kevin R. Elliker, Bethany Hutchings, Anna Wilkinson

**Affiliations:** 1 School of Life and Environmental Sciences, University of Lincoln, Lincoln, UK; 2 Defence Science and Technology Laboratory, Porton Down, Salisbury, UK

**Correction: Animal Cognition (2024) 27:73**

10.1007/s10071-024-01907-0

In this article the ‘Conflict of Interest’ statement was missing and should have been “Anna Wilkinson is an editor for the journal and has not taken part in the manuscript’s peer review process.” The original article has been corrected.

